# Supplementary material for: Iron-sulfur clusters are involved in post-translational arginylation
Source: Nat Commun. 2023 Jan 28;14:458. doi: 10.1038/s41467-023-36158-z (PMC9884297; doi:10.1038/s41467-023-36158-z)
Supplement: Supplementary file 1 — Supplementary Information [file 41467_2023_36158_MOESM1_ESM.pdf]

Supplementary Information

Iron-sulfur clusters are involved in post-translational arginylation

Verna Van[1], Janae B. Brown[1], Corin O'Shea[2], Hannah Rosenbach[3], Ijaz Mohamed[1], Nna-Emeka Ejimogu[1], Toan S. Bui[1], Veronika A. Szalai[4], Kelly N. Chacón[5], Ingrid Span[3], Fangliang Zhang[2,6], and Aaron T. Smith[1*]

[1]Department of Chemistry and Biochemistry, University of Maryland, Baltimore County, Baltimore, Maryland, 21250 USA

[2]Department of Molecular and Cellular Pharmacology, University of Miami, Miller School of Medicine, Miami, Florida, 33136 USA

[3]Institut für Physikalische Biologie, Heinrich-Heine-Universität Düsseldorf, 40225 Düsseldorf, Germany

[4]Physical Measurement Laboratory, National Institute of Standards and Technology, Gaithersburg, Maryland, 20899 USA

[5]Department of Chemistry, Reed College, Portland, Oregon, 97202 USA

[6]Sylvester Comprehensive Cancer Center, University of Miami Miller School of Medicine, Miami, Florida, 33136 USA

[*]To whom correspondence should be addressed. Tel: 410-455-1985; E-mail: smitha@umbc.edu

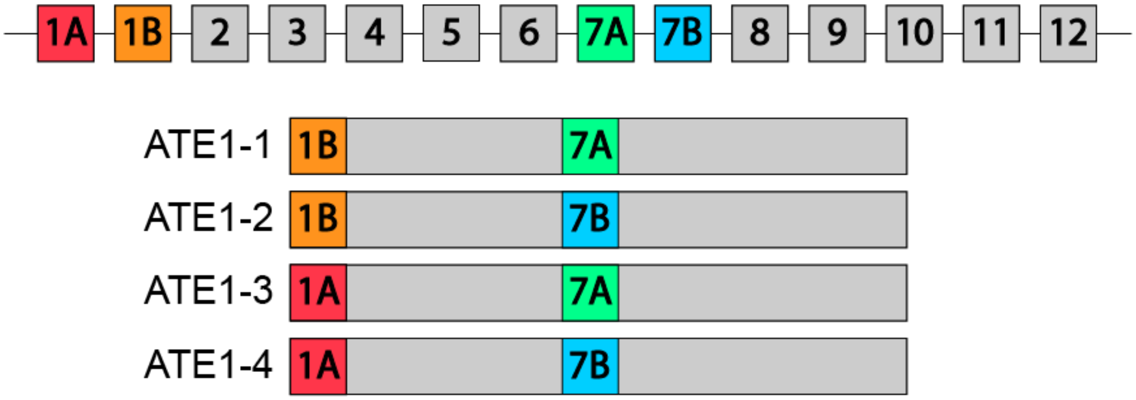

Supplementary Figure 1. The splicing-derived isoforms of *Mus musculus* (*Mm*) ATE1 resulting from alternative splicing of the *Mm ate1* gene. There are two splicing forms of intron 1 (1A,1B) and two splicing forms of intron 7 (7A,7B). The most-commonly studied isoform is that of *Mm*ATE1-1 (formerly known as isoform *Mm*ATE1^{1B7A}).

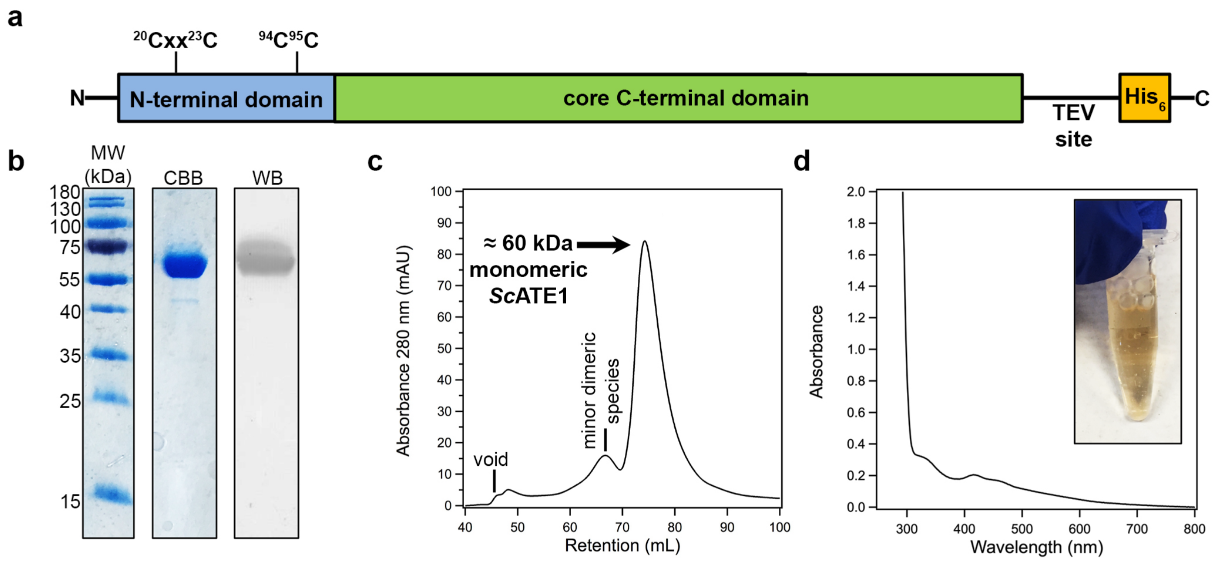

Supplementary Figure 2. Expression, purification, and preliminary characterization of *Saccharomyces cerevisiae* ATE1 (*Sc*ATE1). **a**. Schematic cartoon of the *Sc*ATE1 construct, with the N-terminal domain (site of [Fe-S] cluster binding) in blue, the core C-terminal domain in green, and the C-terminal (His_6) tag in yellow. The expression construct contains all amino acids encoded by the *ate1* gene of *Saccharomyces cerevisiae* (Uniprot identifier P16639). **b**. SDS-PAGE (acrylamide mass fraction of 15 %; CBB) analysis of *Sc*ATE1 post metal-affinity and size-exclusions chromatographies and its respective Western blot (WB). *Sc*ATE1 has an approximate molecular weight of 60000 g/mol (60 kDa; n = 3 independent purifications). **c**. Size-exclusion chromatogram of purified *Sc*ATE1. The major oligomeric species appears to be monomeric based on molecular-weight standards. A minor dimeric species is seen at earlier retention volumes, although this species is variable and comprises < 5 % of our best protein preparations. 1 mAU = 1 absorbance unit/10^{-3}. **d**. Concentrated *Sc*ATE1 expressed in the presence of heme precursors δ-aminolevulinic acid and ferric citrate and purified under oxic conditions displays a brown color (inset), characteristic of [Fe-S] clusters. The electronic absorption spectrum of this purified protein suggests that [Fe-S] clusters are present at low quantities under these expression and purification conditions after size-exclusion chromatography. Source data are provided as a Source Data file.

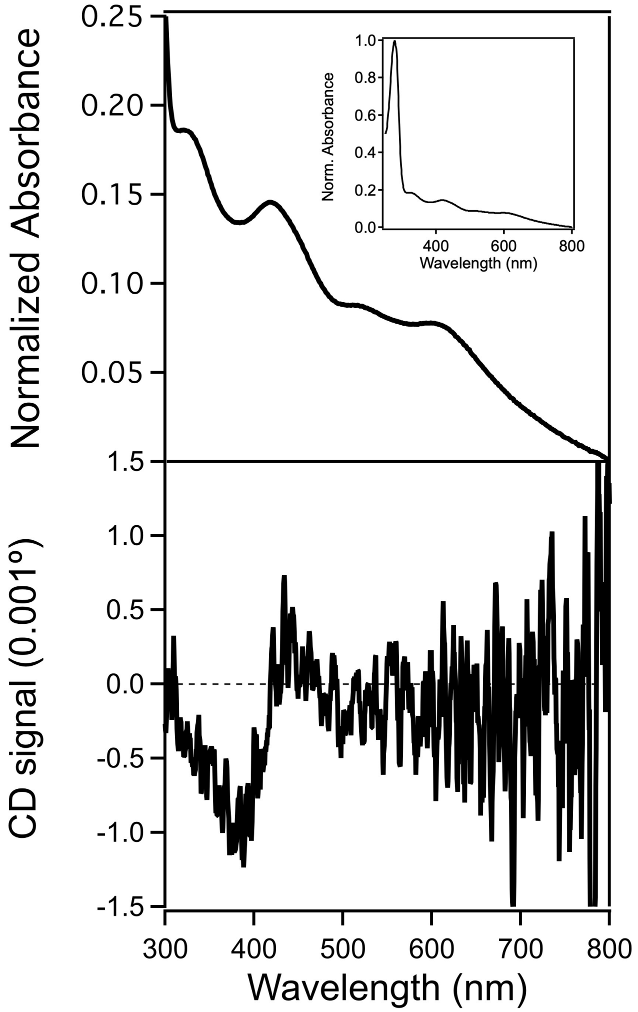

Supplementary Figure 3. The electronic absorption spectrum (top) and circular dichroism (CD) spectrum (bottom) of *Sc*ATE1 expressed in the presence of *L*-Cys, ferric citrate, and iron-sulfur cluster (ISC) biosynthesis machinery, and subsequently purified under oxic conditions. Top, inset: the full electronic absorption spectrum. 0.001° = 1 mdeg. Source data are provided as a Source Data file.

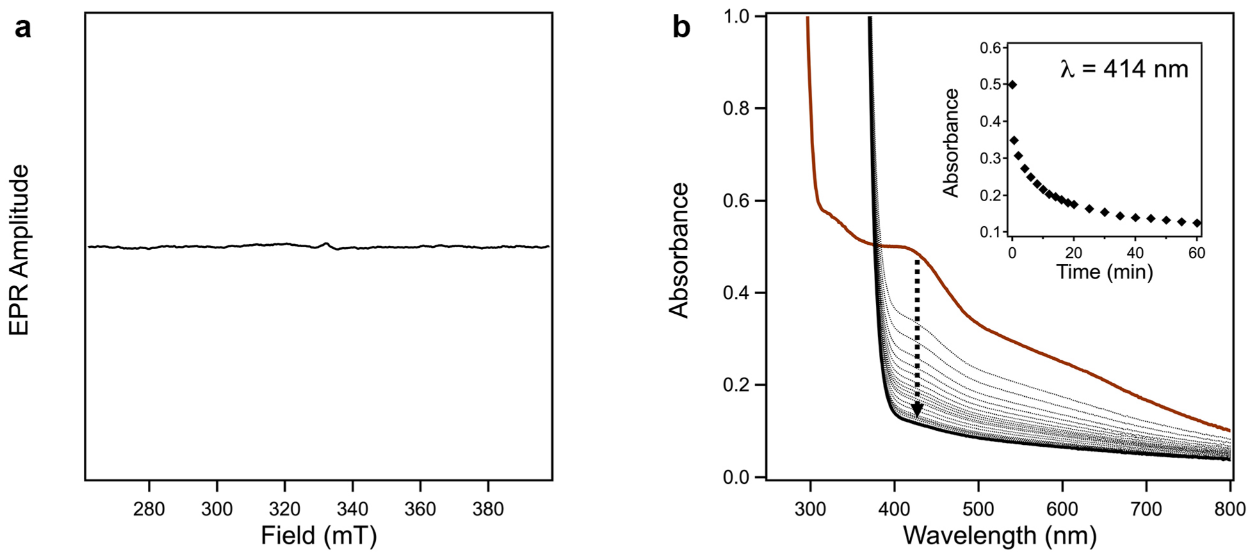

Supplementary Figure 4. Characterization and reactivity of the *Sc*ATE1 [2Fe-2S]$^{2+}$ cluster. **a**. The EPR spectrum of *Sc*ATE1 displays no EPR signal after purification under oxic conditions, consistent with either an antiferromagnetically-coupled [2Fe-2S]$^{2+}$ cluster (supported by other spectral assignments) or an antiferromagnetically-coupled [4Fe-4S]$^{2+}$ cluster (not supported by other spectral assignments under oxic conditions). Spectral acquisition parameters were as follows: frequency = 9.38 GHz, temperature = 20 K, modulation amplitude = 0.5 mT, modulation frequency = 100 kHz, 1024 points, conversion time = 87.89 ms, microwave power = 9.5 mW, 16 scans. For comparison's sake, the spectrum is scaled to the same y-axis as EPR spectra in main text. **b**. Reaction of *Sc*ATE1 [2Fe-2S]$^{2+}$ with sodium dithionite under anoxic conditions results in a time-dependent bleaching (dotted) of the electronic absorption spectrum, from the starting spectrum (brown, solid) to the final spectrum (black, solid). Inset: time-dependent loss of the 414 nm feature of the electronic absorption spectrum of *Sc*ATE1 [2Fe-2S]$^{2+}$after addition of sodium dithionite. Source data are provided as a Source Data file.

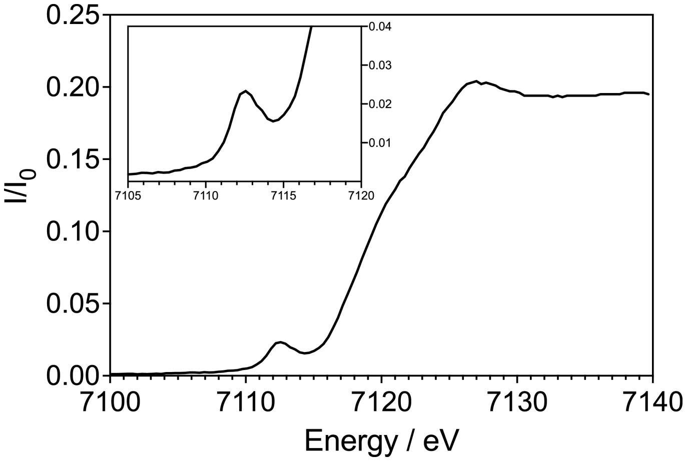

Supplementary Figure 5. The XANES spectrum of *Sc*ATE1 reconstituted under anoxic conditions. Inset: expanded pre-edge feature of the 1s→3d transition at 7112.5 eV, consistent with tetrahedral Fe sites. Source data are provided as a Source Data file.

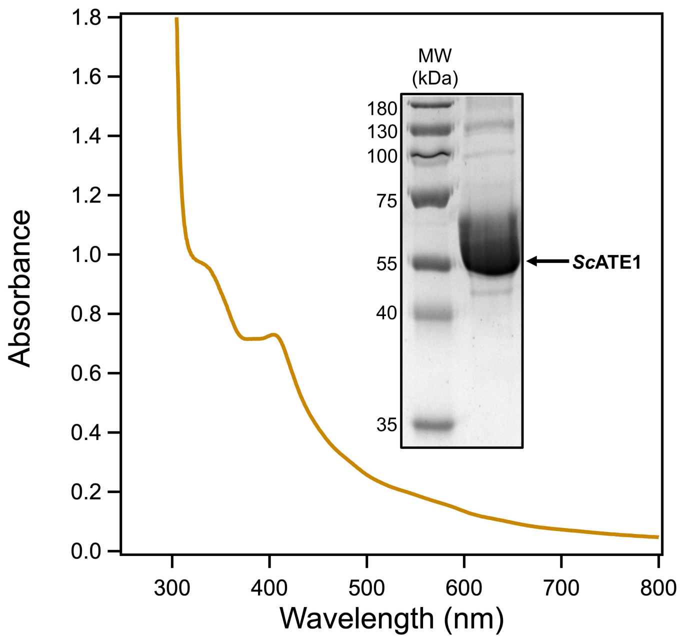

Supplementary Figure 6. *Sc*ATE1 expressed in anaerobic *E. coli* and purified under anoxic conditions binds a higher-order cluster. The electronic absorption spectrum (orange) displays peak maxima indicative of a mixture of $[4Fe\text{-}4S]^{2+}$ and $[3Fe\text{-}4S]^{+}$ clusters. Inset: the SDS-PAGE analysis of *Sc*ATE1 expressed in anaerobic *E. coli* and purified under anoxic conditions demonstrates good final purity, although the total protein yield is significantly diminished. Purified *Sc*ATE1 has an approximate molecular weight of 60000 g/mol (60 kDa). Source data are provided as a Source Data file.

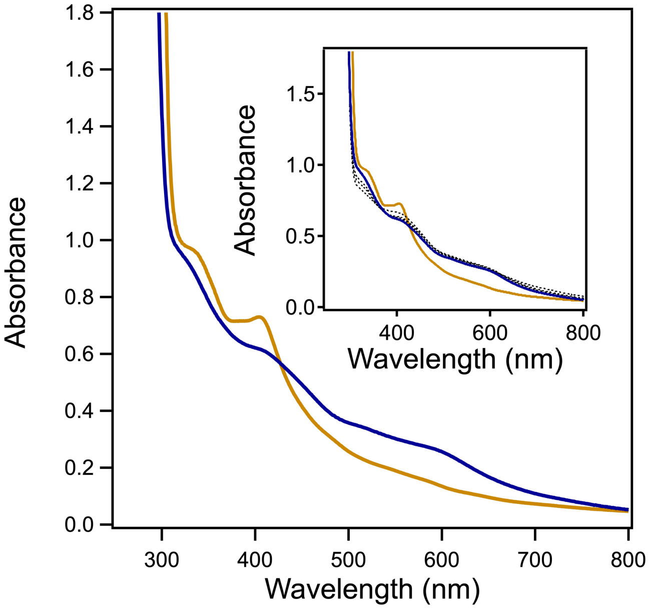

Supplementary Figure 7. *Sc*ATE1 expressed in anaerobic *E. coli* and purified under anoxic conditions is oxygen sensitive. The electronic absorption spectrum of *Sc*ATE1 purified anoxically before (orange) and after (blue) exposure to ambient atmosphere. Inset: time-dependent electronic absorption scans (dotted) of the oxidation of *Sc*ATE1 purified anoxically. The majority of the protein (estimated > 90 %) is oxidized in < 5 min. Source data are provided as a Source Data file.

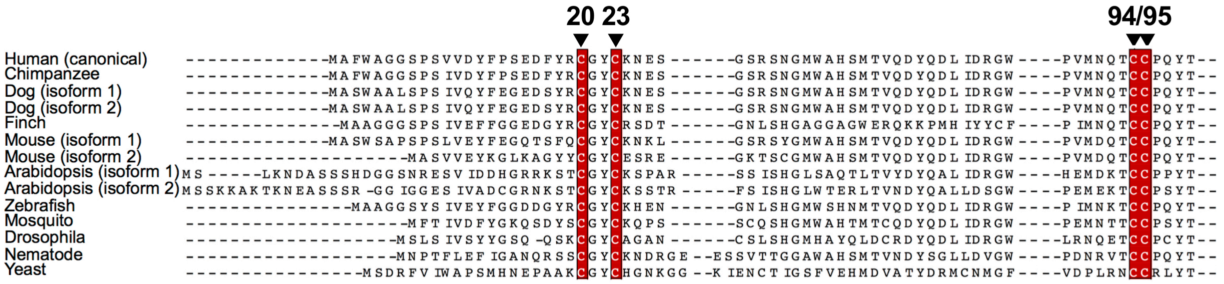

Supplementary Figure 8. Partial protein sequence alignments of the N-terminal domains of eukaryotic ATE1s. For brevity's sake, a small, curated set of exemplary ATE1s across the eukaryotic domain are shown: human (*Homo sapiens*; Uniprot ID: P16639), chimpanzee (*Pan troglodytes*; Uniprot ID: H2Q2P4), dog (*Canus familiaris*; isoform 1 Uniprot ID: J9P4X6; isoform 2 Uniprot ID: E2RS45), finch (*Poephila guttata*; Uniprot ID: H0ZM06), mouse (*Mus musculus*; isoform 1 Uniprot ID: Q80YP1; isoform 2 Uniprot ID: Q4FCQ6), arabidopsis (*Arabidopsis thaliana*; isoform 1 Uniprot ID: Q9ZT48; isoform 2 Uniprot ID: Q9C776), zebrafish (*Brachydanio rerio*; Uniprot ID: G8XPI0), mosquito (*Aedes aegypti*; Uniprot ID: Q178G8), drosophila (*Drosophila melanogaster*; Uniprot ID: O96539), nematode (*Caenorhabditis elegans*; Uniprot ID: P90914), and yeast (*Saccharomyces cerevisiae*; Uniprot ID: P16639). The wholly-conserved Cys residues involved in cluster binding are highlighted in red; the numbering of the Cys residues at the top of the figure is based on the yeast (*Sc*ATE1) protein sequence.

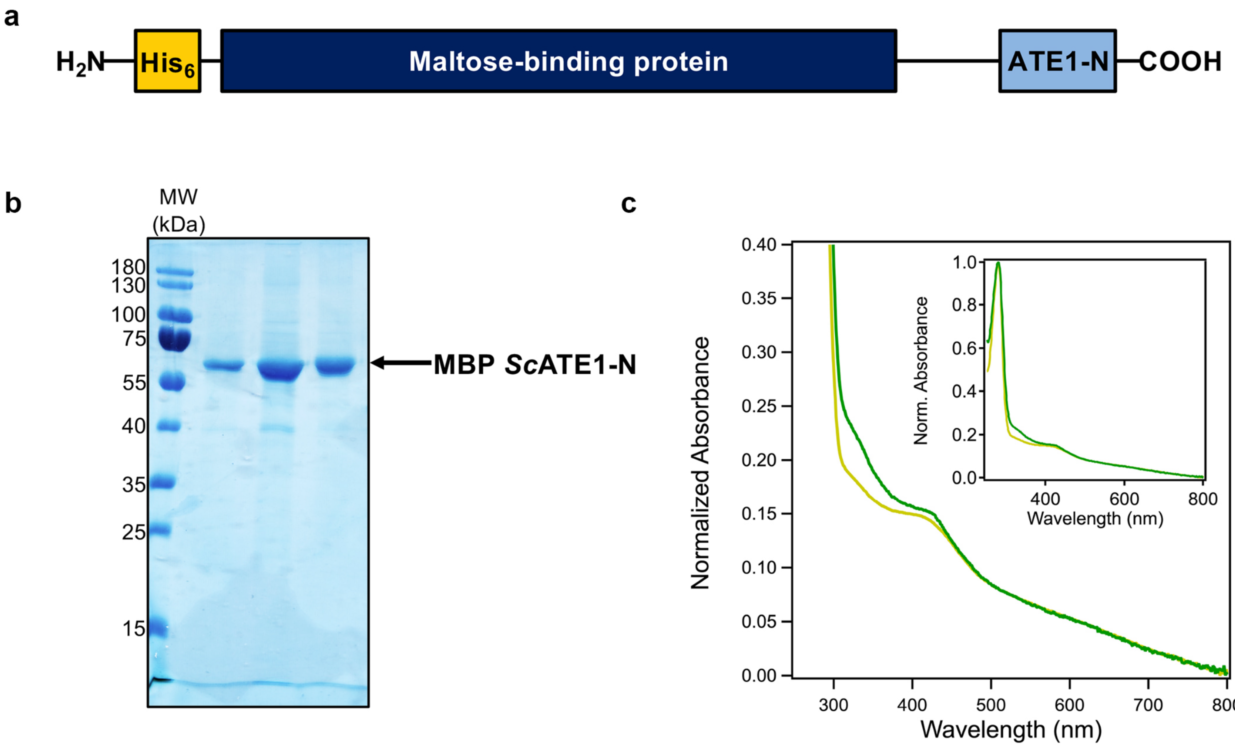

Supplementary Figure 9. Features of the MBP *Sc*ATE1-N construct. **a**. Schematic cartoon of the MBP *Sc*ATE1-N construct. The construct consists of an N-terminal $(His)_6$ tag (for orthogonal purification and Western blotting, yellow box), a maltose-binding protein segment for solubility (dark blue box), and the N-terminal 146 amino acids of *Sc*ATE1 (light blue box). **b**. Representative SDS-PAGE (acrylamide mass fraction of 15 %) analysis of MBP *Sc*ATE1-N (n = 3 independent purifications) post amylose column. MBP *Sc*ATE1-N has an approximate molecular weight of 61100 g/mol (61.1 kDa). **c**. Overlay of the electronic absorption spectrum of WT *Sc*ATE1 reconstituted under anoxic conditions (yellow) and MBP *Sc*ATE1-N also reconstituted under anoxic conditions (green). Inset: the full spectra color-coded the same way. The spectral are almost identical, indicating that the reconstituted $[4Fe\text{-}4S]^{2+}$ cluster is bound in the *Sc*ATE1 N-terminal domain. Source data are provided as a Source Data file.

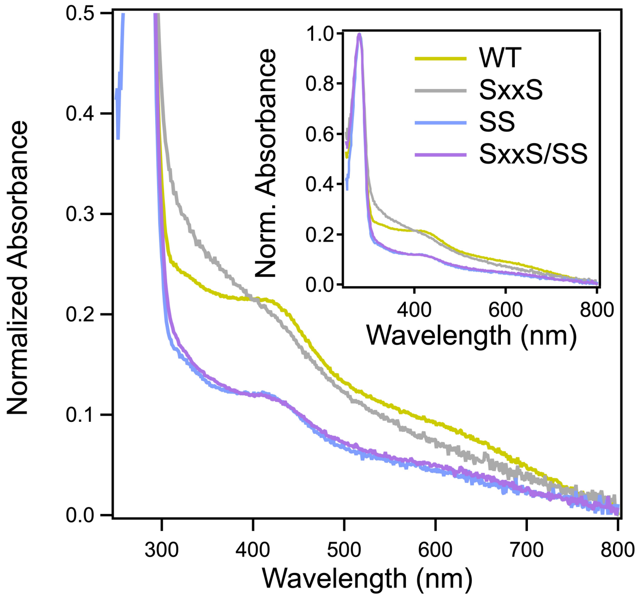

Supplementary Figure 10. Overlay of the electronic absorption spectra of WT *Sc*ATE1 (yellow), variant $S^{20}xxS^{23}$ *Sc*ATE1 (gray), variant $S^{94}S^{95}$ *Sc*ATE1 (blue), and variant $S^{20}xxS^{23}/S^{94}S^{95}$ *Sc*ATE1 (purple) all reconstituted under anoxic conditions. All variants have distinct and/or diminished electronic absorption spectra compared to the WT protein. The largest impact in cluster binding appears to alteration of the $C^{20}xxC^{23}$ motif to $S^{20}xxS^{23}$. Some adventitious iron binding is clear even in the quadruple variant, but the spectrum is still dramatically distinct from the WT protein. Source data are provided as a Source Data file.

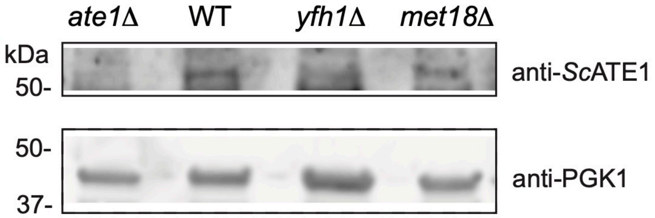

Supplementary Figure 11. Representative Western Blot images (*n* = 2 replicates and verified in Ref. [70]) showing the levels of endogenous ATE1 in *Saccharomyces cerevisiae yfh1*Δ and *met18*Δ, in comparison to the wild-type and *ate1*Δ yeasts, which serve as positive and negative control, respectively. The level of PGK1 was used for a loading control. Source data are provided as a Source Data file.

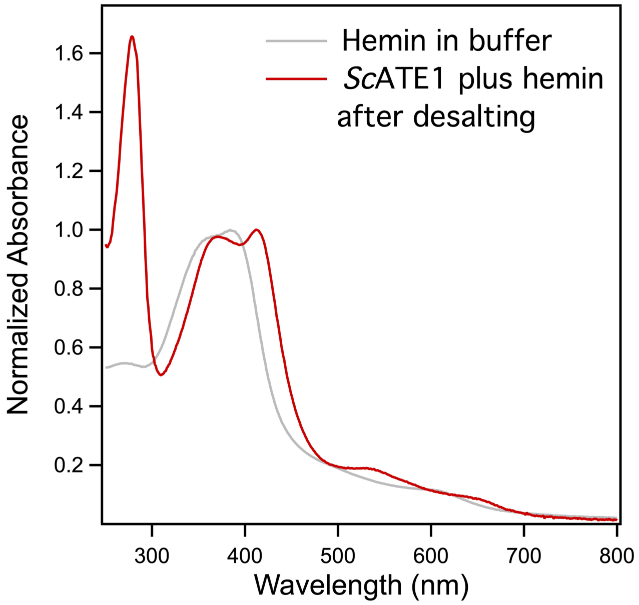

Supplementary Figure 12. *Sc*ATE1 with titrated hemin has an electronic absorption spectrum consistent with non-specific heme binding. The electronic absorption spectrum of heme added to *Sc*ATE1 is shown (red) after a desalting column to remove any unbound heme. For reference, hemin dissolved in *Sc*ATE1 size-exclusion buffer (see *Methods* section) is shown in gray. Source data are provided as a Source Data file.

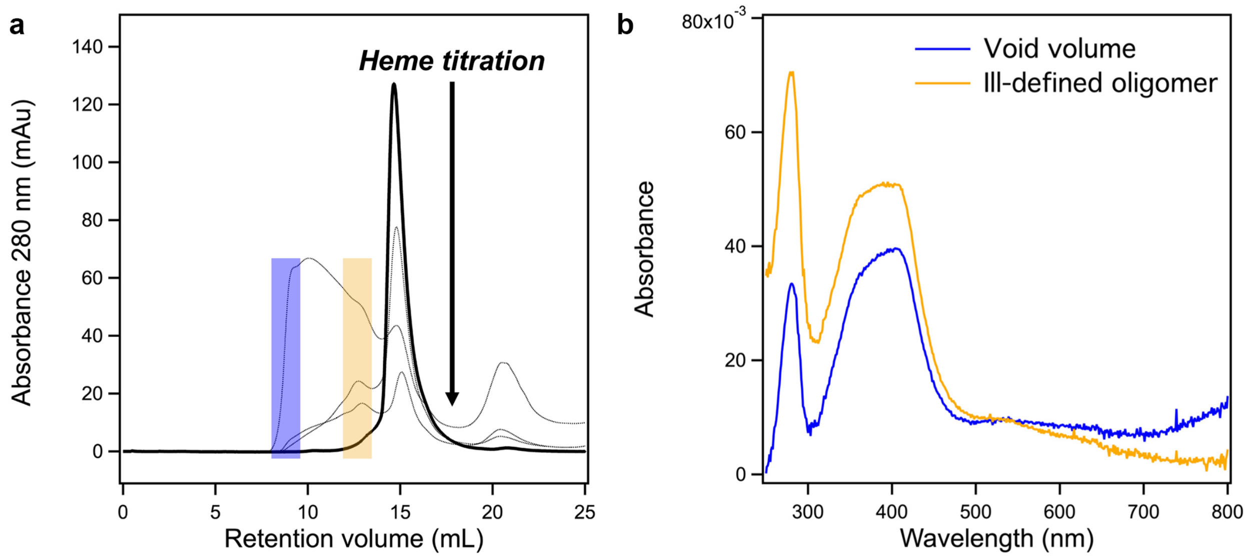

Supplementary Figure 13. Titration of heme into *Sc*ATE1 causes protein aggregation. **a**. In the apo form, *Sc*ATE1 is monodisperse and monomeric in solution (black solid trace) based on its retention volume (*ca*. 15 mL) compared to external standards. As heme is titrated into the protein (1, 2, 3, and 5 mole equivalents; gray dotted traces), the monodisperse and monomeric species is lost, resulting in an inactive ill-defined oligomer (*ca*. 12 mL retention volume) and inactive aggregate (*ca*. 9 mL retention volume; *i.e.*, the void volume of the column). 1 mAU = absorbance unit/10^{-3}. **b**. After titration and size-exclusion chromatography, the only species that have appreciable heme bound to them are those of the inactive ill-defined oligomer (electronic absorption spectrum in orange) and the inactive aggregated species (electronic absorption spectrum in blue). Highlighted on panel **a**. are the fractions that were collected to generate the electronic absorption spectra in panel **b**. Source data are provided as a Source Data file.

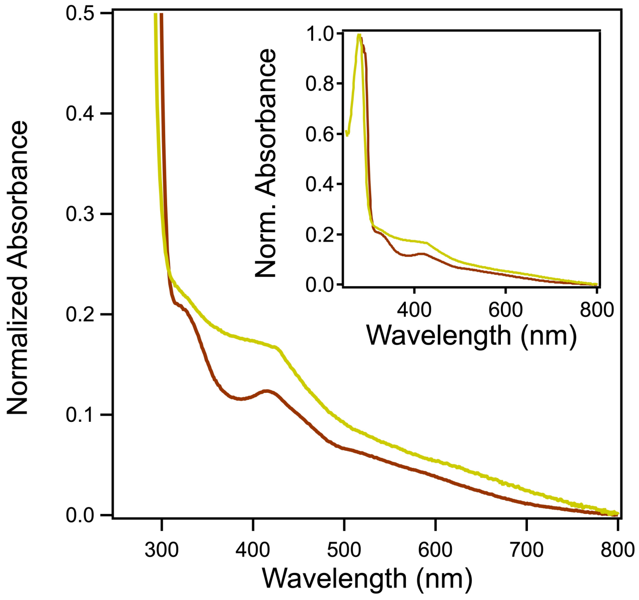

Supplementary Figure 14. Mouse ATE1 (*Mm*ATE1-1) is also an [Fe-S] cluster-binding protein. Expression of *Mm*ATE1-1 in the presence of ferric citrate and *L*-Cys and subsequent purification under oxic conditions yields an electronic absorption spectrum (brown trace) indicative of a [2Fe-2S] cluster, nearly identical to *Sc*ATE1. Apo purified *Mm*ATE1-1 reconstituted under anoxic conditions yields an electronic absorption spectrum (yellow trace) indicative of a [4Fe-4S] cluster, nearly identical to *Sc*ATE1 and MBP *Sc*ATE1-N. Inset: the full spectra color-coded the same way as the main figure. Source data are provided as a Source Data file.

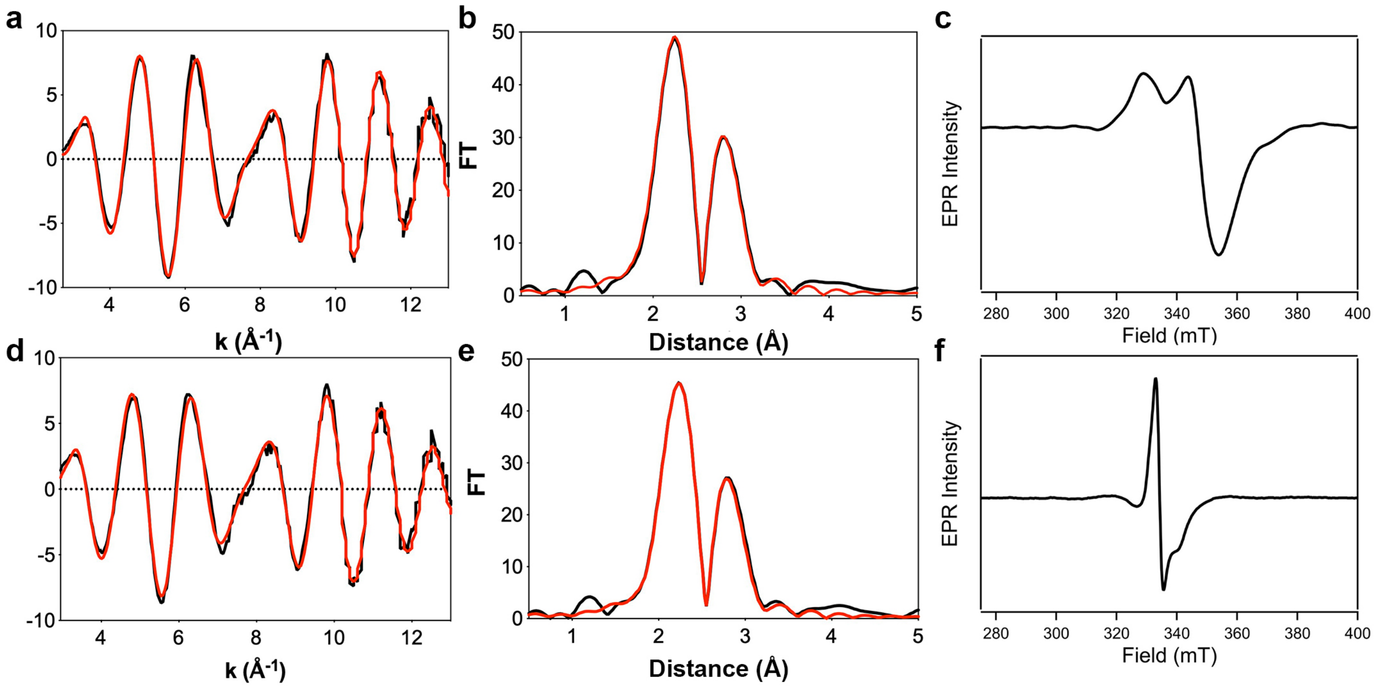

Supplementary Figure 15. *Mm*ATE1-1 is also an O_2-sensitive [Fe-S] cluster-binding protein. **a,b**. EXAFS of *Mm*ATE1-1 reconstituted under anoxic conditions. The raw EXAFS (**a**) and the Fourier transformed data (**b**) are consistent with the presence of a [4Fe-4S] cluster. The black traces represent the experimental data while the red traces represent the EXCURVE-fitted data. 1 Å = 0.1 nm. **c**. A $[4Fe\text{-}4S]^+$ cluster is clearly observed in the dithionite-reduced EPR spectrum (black) of anoxically-reconstituted *Mm*ATE1-1. Conditions were similar to those in Figure 3 with the following exceptions: temperature = 10 K and 128 scans. **d,e**. EXAFS of *Mm*ATE1-1 reconstituted under anoxic conditions after exposure to O_2. The raw EXAFS (**d**) and the Fourier transformed data (**e**) are most consistent with a mixture of a [3Fe-4S] cluster and a [2Fe-2S] cluster. The black traces represent the experimental data while the red traces represent the EXCURVE-fitted data. 1 Å = 0.1 nm. **f**. After anoxic reconstitution of *Mm*ATE1-1, reaction with O_2, and desalting, a $[3Fe\text{-}4S]^+$ cluster is readily apparent. Conditions were similar to those in Figure 3 with the following exceptions: temperature = 20 K, microwave power = 5 mW, and 16 scans. Source data are provided as a Source Data file.

Supplementary Table 1. Fits obtained for the Fe K-EXAFS *Mm*ATE1 reconstituted anoxically and containing a [4Fe-4S] cluster, and *Mm*ATE1 reconstituted anoxically and exposed to O_2, in which the cluster converts to a mixed [3Fe-4S] and [2Fe-2S] species. Fits were determined by curve fitting using the program EXCURVE (version 9.2).

		**Fe-S**			**Fe-Fe**			**Fe-Fe**			**E_o[e] (eV)**
**Sample**	**Fit index[a]**	**No[b]**	**R[c] (Å)[f]**	**DW[d] (Å^2)**	**No**	**R (Å)**	**DW (Å^2)**	**No**	**R (Å)**	**DW (Å^2)**	
***Mm*ATE1 reconstituted anoxically**	0.14	4	2.26	0.011	2	2.71	0.004	1	2.53	0.011	-2.18
***Mm*ATE1 reconstituted anoxically and exposed to O_2**	0.13	3.5	2.25	0.009	1.5	2.71	0.003	0.5	2.55	0.005	-2.79

[a]The least-squares fitting parameter (see *Methods*) [b]Coordination number [c]Bond length [d]Debye-Waller factor [e]Photoelectron energy threshold [f]1 Å = 0.1 nm

Supplementary Table 2. List of primers used in this study for the mutagenesis of *Sc*ATE1 produced recombinantly in *E. coli*, and primers used for the generation of *Saccharomyces cerevisiae* strain BY4741 (referred to as yeast for simplicity) *ate1* mutants for cell-stress assays.

Primer name	Primer sequence
WT $C^{20}xxC^{23}$ to $S^{20}xxS^{23}$ forward	5'-CCGTGGCTGTAACCGCTTTTCGCCGCCGGC-3'
WT $C^{20}xxC^{23}$ to $S^{20}xxS^{23}$ reverse	5'-GCCGGCGGCGAAAAGCGGTTACAGCCACGG-3'
WT $C^{94}C^{95}$ to $S^{94}S^{95}$ forward	5'-TGGTATACAGACGGCTGCTGTTACGCAGCGGATC-3'
WT $C^{94}C^{95}$ to $S^{94}S^{95}$ reverse	5'-GATCCGCTGCGTAACAGCAGCCGTCTGTATACCA-3'
Variant $S^{20}xxS^{23}$; $C^{94}C^{95}$ to $S^{94}S^{95}$ forward	5'-TGGTATACAGACGGCTGCTGTTACGCAGCGGATC-3'
Variant $S^{20}xxS^{23}$; $C^{94}C^{95}$ to $S^{94}S^{95}$ reverse	5'-GATCCGCTGCGTAACAGCAGCCGTCTGTATACCA-3'
Yeast ATE1 6xHis upstream primer	5’-ATATAAGCTTATGAGCGACCGTTTCGTG-3’
Yeast ATE1 6xHis downstream primer	5’-ATATTCTAGATCAGTGGTGGTGGTGGTGGTGCTCGAGGCTCTG-3’
