# Supplementary material for: Iron-sulfur clusters are involved in post-translational arginylation
Source: Nat Commun. 2023 Jan 28;14:458. doi: 10.1038/s41467-023-36158-z (PMC9884297; doi:10.1038/s41467-023-36158-z)
Supplement: Supplementary file 3 — Reporting Summary [file 41467_2023_36158_MOESM3_ESM.pdf]

nature portfolio

Corresponding author(s): Aaron T. Smith

Last updated by author(s): Jan 16, 2023

Reporting Summary

Nature Portfolio wishes to improve the reproducibility of the work that we publish. This form provides structure for consistency and transparency in reporting. For further information on Nature Portfolio policies, see our Editorial Policies and the Editorial Policy Checklist.

Statistics

For all statistical analyses, confirm that the following items are present in the figure legend, table legend, main text, or Methods section.

n/a	Confirmed	
☐	☒	The exact sample size (*n*) for each experimental group/condition, given as a discrete number and unit of measurement
☐	☒	A statement on whether measurements were taken from distinct samples or whether the same sample was measured repeatedly
☐	☒	The statistical test(s) used AND whether they are one- or two-sided<br>*Only common tests should be described solely by name; describe more complex techniques in the Methods section.*
☒	☐	A description of all covariates tested
☐	☒	A description of any assumptions or corrections, such as tests of normality and adjustment for multiple comparisons
☐	☒	A full description of the statistical parameters including central tendency (e.g. means) or other basic estimates (e.g. regression coefficient) AND variation (e.g. standard deviation) or associated estimates of uncertainty (e.g. confidence intervals)
☐	☒	For null hypothesis testing, the test statistic (e.g. *F*, *t*, *r*) with confidence intervals, effect sizes, degrees of freedom and *P* value noted<br>*Give P values as exact values whenever suitable.*
☒	☐	For Bayesian analysis, information on the choice of priors and Markov chain Monte Carlo settings
☒	☐	For hierarchical and complex designs, identification of the appropriate level for tests and full reporting of outcomes
☒	☐	Estimates of effect sizes (e.g. Cohen's *d*, Pearson's *r*), indicating how they were calculated

Our web collection on statistics for biologists contains articles on many of the points above.

Software and code

Policy information about availability of computer code

Data collection	No custom algorithms or software were used for data collection.
Data analysis	EXAFSPAK (Microsoft Windows version), EXCURVE (version 9.2), IgorPro (version 6.0), JalView (version 2.7), ClustalW algorithm (version 2.1) implementing the Blosum62 matrix

For manuscripts utilizing custom algorithms or software that are central to the research but not yet described in published literature, software must be made available to editors and reviewers. We strongly encourage code deposition in a community repository (e.g. GitHub). See the Nature Portfolio guidelines for submitting code & software for further information.

Data

Policy information about availability of data

All manuscripts must include a data availability statement. This statement should provide the following information, where applicable:

- Accession codes, unique identifiers, or web links for publicly available datasets
- A description of any restrictions on data availability
- For clinical datasets or third party data, please ensure that the statement adheres to our policy

Source data are provided with this paper. The data generated in this study are provided in the Supplementary Information/Source Data file. The following Uniprot IDs were used in this study: Homo sapiens ATE1, A0A8I5KT53; Pan troglodytes ATE1, H2Q2P4; Canus familiaris ATE1, A0A8I3PC88 and A0A8I3PH57; Poephila guttata ATE1, H0ZM06; Mus musculus ATE1, Q80YP1 and Q4FCQ6; Arabidopsis thaliana ATE1, Q9ZT48 and Q9C776; Brachydanio rerio ATE1, G8XPI0; Aedes aegypti ATE1, Q178G8; Drosophila melanogaster ATE1, O96539; Caenorhabditis elegans ATE1, P90914; Saccharomyces cerevisiae ATE1, P16639; Escherichia coli maltose binding

protein, P0AEX9; and Escherichia coli ArgRS, P11875. All unique biological materials (e.g., expression plasmids) are readily available from the authors upon request.

Human research participants

Policy information about studies involving human research participants and Sex and Gender in Research.

Reporting on sex and gender	This study did not involve human research participants; therefore, reporting on sex and gender is not applicable.
Population characteristics	This study did not involve human research participants; therefore, reporting on population characteristics is not applicable.
Recruitment	This study did not involve human research participants; therefore, reporting on recruitment is not applicable.
Ethics oversight	This study did not involve human research participants; therefore, reporting on ethics oversight is not applicable.

Note that full information on the approval of the study protocol must also be provided in the manuscript.

Field-specific reporting

Please select the one below that is the best fit for your research. If you are not sure, read the appropriate sections before making your selection.

[x] Life sciences [ ] Behavioural & social sciences [ ] Ecological, evolutionary & environmental sciences

For a reference copy of the document with all sections, see nature.com/documents/nr-reporting-summary-flat.pdf

Life sciences study design

All studies must disclose on these points even when the disclosure is negative.

Sample size	All sample sizes were chosen to be at least three independent trials or greater in order to derive statistical significance.
Data exclusions	No data were excluded from the analyses.
Replication	Data in which average values are noted (in vitro arginylation assays, yeast stress assays, and in vivo yeast arginylation assays) represent experiments of at least three independent trials and are reported as the mean +/- one standard deviation of the mean. All attempts at replication were successful.
Randomization	No randomization was performed as randomization was unnecessary to assess the presence of the [Fe-S] cofactor on in vitro and in vivo arginylation.
Blinding	The investigators were not blinded, which is not relevant to assessing the presence of the [Fe-S] cofactor on in vitro and in vivo arginylation, as blinding is more appropriate for clinical trials.

Reporting for specific materials, systems and methods

We require information from authors about some types of materials, experimental systems and methods used in many studies. Here, indicate whether each material, system or method listed is relevant to your study. If you are not sure if a list item applies to your research, read the appropriate section before selecting a response.

Materials & experimental systems

n/a	Involved in the study	
[ ]	[x]	Antibodies
[ ]	[x]	Eukaryotic cell lines
[x]	[ ]	Palaeontology and archaeology
[x]	[ ]	Animals and other organisms
[x]	[ ]	Clinical data
[x]	[ ]	Dual use research of concern

Methods

n/a	Involved in the study	
[x]	[ ]	ChIP-seq
[x]	[ ]	Flow cytometry
[x]	[ ]	MRI-based neuroimaging

Antibodies

Antibodies used	Anti-poly Histidine-Peroxidase antibody, purchased from Millipore-Sigma catalog #: A7058-1VL; beta Actin N-terminal arginylation antibody, custom ordered from Genscript; mCherryFP antibody, clone 16D7 purchased from ThermoScientific.

Validation	Anti-poly Histidine-Peroxidase antibody was validated commercially by Millipore-Sigma (catalog #: A7058-1VL); beta Actin N-terminal arginylation antibody was validated in Kumar, A. et al. Posttranslational arginylation enzyme Ate1 affects DNA mutagenesis by regulating stress response. Cell death & disease 7, e2378, doi:10.1038/cddis.2016.284; mCherryFP antibody clone 16D7 validation can be found on the manufacturer's website, SKU: M11217

Eukaryotic cell lines

Policy information about cell lines and Sex and Gender in Research

Cell line source(s)	Open Biosystems (ate1 deletion, catalog ID YSC6273-201936070; met18 deletion, catalog ID YSC6273-201925518; yfh1 deletion, catalog ID YSC6274-201926392)
Authentication	Authentication performed as described in Kumar, A.; Birnbaum, M. D.; Patel D. M.; Morgan, W. H.; Singh, J.; Barrientos, A.; Zhang, F. Posttranslational arginylation enzyme Ate1 affects DNA mutagenesis by regulating the stress response. Cell Death and Disease 2016, 7, e2378.
Mycoplasma contamination	Cells were not tested for mycoplasma contamination.
Commonly misidentified lines (See ICLAC register)	There is no commonly misidentified cell line.
